# Statistical Determination of Rainfall-Runoff Erosivity Indices for Single Storms in the Chinese Loess Plateau

**DOI:** 10.1371/journal.pone.0117989

**Published:** 2015-03-17

**Authors:** Mingguo Zheng, Xiaoan Chen

**Affiliations:** 1 Key Laboratory of Water Cycle and Related Land Surface Processes, Institute of Geographic Sciences & Natural Resources Research, Chinese Academy of Sciences, Beijing 100101, China; 2 Jiangxi Institute of Soil and Water Conservation, Nanchang 330029, China; Centro de Investigacion Cientifica y Educacion Superior de Ensenada, MEXICO

## Abstract

Correlation analysis is popular in erosion- or earth-related studies, however, few studies compare correlations on a basis of statistical testing, which should be conducted to determine the statistical significance of the observed sample difference. This study aims to statistically determine the erosivity index of single storms, which requires comparison of a large number of dependent correlations between rainfall-runoff factors and soil loss, in the Chinese Loess Plateau. Data observed at four gauging stations and five runoff experimental plots were presented. Based on the Meng’s tests, which is widely used for comparing correlations between a dependent variable and a set of independent variables, two methods were proposed. The first method removes factors that are poorly correlated with soil loss from consideration in a stepwise way, while the second method performs pairwise comparisons that are adjusted using the Bonferroni correction. Among 12 rainfall factors, *I*_30_ (the maximum 30-minute rainfall intensity) has been suggested for use as the rainfall erosivity index, although *I*_30_ is equally correlated with soil loss as factors of *I*_20_, *EI*_10_ (the product of the rainfall kinetic energy, *E*, and *I*_10_), *EI*_20_ and *EI*_30_ are. Runoff depth (total runoff volume normalized to drainage area) is more correlated with soil loss than all other examined rainfall-runoff factors, including *I*_30_, peak discharge and many combined factors. Moreover, sediment concentrations of major sediment-producing events are independent of all examined rainfall-runoff factors. As a result, introducing additional factors adds little to the prediction accuracy of the single factor of runoff depth. Hence, runoff depth should be the best erosivity index at scales from plots to watersheds. Our findings can facilitate predictions of soil erosion in the Loess Plateau. Our methods provide a valuable tool while determining the predictor among a number of variables in terms of correlations.

## Introduction

Rainfall erosivity indicates the potential of a storm to erode soil. A single index of rainfall erosivity that can measure the composite effect of various rainstorm characteristics on soil erosion is highly desirable for predicting soil loss [1, 2]. It is well known that soil losses are frequently due to a few intense rainfall events [1, 3]. The most common erosivity index for single storms is the *EI*_30_ index (the product of the rainfall kinetic energy, *E*, and the maximum 30-min intensity, *I*_30_), as is used in the Universal Soil Loss Equation (USLE) [4] and in the Revised Universal Soil Loss Equation (RUSLE) [5]. The calculation of *EI*_30_ is of high data requirements and labor intensive [2, 6]. For this reason, a large number of studies (e.g. [1, 2, 6, 7]) were devoted to developing a proxy, using more readily available data such as daily, monthly and annual precipitations, for *EI*_30_ and the *R* factor of the USLE (the mean annual total of *EI*_30_).

Besides the *EI*_30_ index, other forms of erosivity index for storm events primarily include the *KE* > 25 index (the total kinetic energy for rainfall duration with intensity exceeding 25 mm h^-1^) [8], the *PI*_m_ index (the product of the rainfall amount, *P*, and the peak rainfall intensity, *I*_m_) [9], the *I*_x_ *E*_A_ index (the product of the excess rainfall rate, *I*_x_, and the rainfall kinetic energy flux, *E*_A_) [10], and the so-called *A* index [11]. Many local erosivity indices for single storms have also been used, such as *I*15 in Belgium [12], *EI*5 in NE Spain [13], *E* in Palestinian areas [14] and *EI*_60_ and *I*60 in Malaysia [15]. In the Chinese Loess Plateau, both *Wang* [16] and *Jia et al*. [17] suggested *EI*_10_ as the rainfall erosivity index; however, *EI*_10_ has been shown to be of similar effectiveness as *EI*_30_ [18]. Notably, *Chen et al*. [19] detected no significant difference among correlations between soil loss and a set of *EI*_t_ variables (*EI*_10_, *EI*_20_, *EI*_30_, *EI*_40_, *EI*_50_ and *EI*_60_). Furthermore, it was found that *EI*_t_ did not greatly improve soil loss predictions compared with *PI*_t_ [19–21], which can thus serve as a surrogate for *EI*_30_.

Both raindrops and runoff are drivers of soil erosion [22]. Runoff factors, mainly runoff volume and peak discharge, have frequently been included into an erosivity index [23–28]. The addition of runoff terms can improve the ability of models to predict soil loss or sediment yield especially for small to medium events [25, 27, 29]. Foster et al. [25] found that the lumped erosivity factors including rainfall amount, rainfall intensity and runoff amount performs better than *EI*_30_, whereas erosivity factors with separate terms for rainfall and runoff erosivity performs best. However, Foster et al. [25] acknowledged their inability to determine whether the observed improvements were statistically significant or not. In the Modified Universal Soil Loss Equation (MUSLE), the *EI*_30_ index is replaced by a power of the product of runoff volume and peak discharge [24], as is also used in the Agricultural Policy/Environmental eXtender (APEX) model [28]. In another modified version of the USLE called the USLE-M [27], the erosivity index of single events is the product of *EI*_30_ and the runoff ratio. In the Loess Plateau, many studies included both terms of runoff volume and peak discharge, in a lumped or separated form, into models for predicting soil loss or sediment yield [30–32]. Nevertheless, it is well known that runoff volume alone can adequately predict sediment yield of the flood event in the Loess Plateau (*r* ^2^ > 0.9) [33]. A proportional model of event runoff volume and sediment yield applies well over a wide range of spatial scales from hill slopes to large-sized watersheds [34–35].

To determine the erosivity index, a large number of correlations between rainfall-runoff factors and soil loss often need to be compared (e.g. [14–17, 23, 25, 26, 36–37]). For example, the *EI*_30_ index was established as the rainfall erosivity index of the USLE by comparing correlations of more than 40 factors with soil loss [38–40]. As a result of the indelible sampling error, sample correlation coefficients can never be identical to population ones. Because the sample difference does not fully represent the population difference, a statistical test is needed to determine the significance of the observed sample difference. To our knowledge, no studies have applied statistical tests while determining the erosivity index with the exceptions of [12] and [19], although a number of statistical tests for comparing dependent or independent correlations exist [41].

The object of this study is to determine erosivity indices for single storms on a statistical basis using data observed in the Chinese Loess Plateau. After describing the study area and data source, we present two methods that compare a large number of dependent correlations. The two methods build on the Meng’s tests [42], which has been widely used to compare correlations in psychological research. We then determine the rainfall-runoff erosivity indices among a large number of factors using the two methods. We finally made some discussions about rainfall and runoff factors with an emphasis on the best erosivity index in the Loess Plateau.

## Study Area and Data

The present study uses data observed at 5 runoff experimental plots and 4 gauging stations (Tables 1 and 2) within the Dalihe River watershed (See Fig. 1 in [35] for the location), a secondary-order river of the middle Yellow River. Typical of the Loess Plateau, the loess mantle of the Dalihe watershed is generally thicker than 100 m. The climate is typically semiarid with a mean annual precipitation of 440 mm (1960–2002). Soil erosion is primarily caused by localised short-duration, high-intensity convective rainstorms. A single storm can commonly cause a soil loss of greater than 10 000 t km^-2^. Most of the lands were intensively cultivated with little soil conservation practices during the monitoring period (1959–1969). The terrain is very precipitous and deeply dissected.

**Table 1:** The gauging stations in the Dalihe River watershed.

Station No.^a^	Creek/River	Gauging station	Area (km^2^)	Data Period	*n* ^b^
3	Tuanshangou	Tuanshangou	0.18	1961–69	44
4	Shejiagou	Shejiagou	4.26	1960–69	49
9	Chabagou	Caoping	187	1959–69	64
12	Dalihe	Suide	3893	1960–69	44

^a^ The station numbers correspond to those given in Fig. 1(b) in [35].

^b^ *n* is the number of recorded flood events.

**Table 2:** The runoff experimental plots in the Tuanshangou subwatershed.^a^

Plot	Slope length (m)	Slope (°)	Horizontal area (m^2^)	Data period	*n* ^b^
Plot 4	20	22	300	1963–67	25
Plot 2	40	22	600	1963–67	27
Plot 3	60	22	900	1961–69	45
Plot 7	126	32	5740	1961–69	40
Plot 9	164	27	17200	1963–69	41

^a^ The layouts of the experimental plots were specified in Fig. 1(c) in [35].

^b^ *n* is the number of recorded storm events.

All examined plots are located within the Tuanshangou subwatershed (latitude 37°41′N, longitude 109°58′E; See Fig. 1(c) in [35] for the location), a headwater basin of the Dalihe watershed. The plots were all under arable with crops varying between years, generally including millet, potato, mung bean, clover, sorghum and wheat. The vegetation cover rarely exceeded 25% in the plots. The recorded maximum *I*_10_ is 2.17 mm min^-1^ (1961–1969). Rill erosion is dominant on upland slopes. During the 1960s, the annual erosion intensity, averaging 41 000 t km^-2^ at Plots 4, 2 and 3 (Table 2), was maximized in 1966. Although rills only occurred on 5 out of 54 rainfall days, these five days contributed almost all of the annual soil loss (>96%) in 1966. Downslope, the valley side slope is generally very steep (>35°), allowing the emergence of permanent, incised gullies and mass wasting events.

Unless stated otherwise, all data used in this study were obtained from the Yellow River Water Conservancy Commission (YRWCC). The YRWCC stream-gauging crews conducted all measurements. Hyetograph data were obtained using a rainfall gauge near Plot 3 (See Fig. 1(c) in [35] for the location). The observation interval was generally smaller than 10 min, even 1 min in many cases of high rainfall intensity. The field monitoring programs of runoff and sediment have been described in detail in [43, 44].

Based on the instantaneous measurement of water discharge and sediment concentration, the runoff depth, *h* (mm: total runoff volume normalized to drainage area), and the specific sediment yield, *SSY* (t km^−2^) of single events were calculated. Conventionally, the term “soil erosion” is used for hill slopes, and the term “sediment yield” is used for a river system or watershed. For simplicity, we use *SSY* to represent both cases hereafter. The event mean sediment concentration, *SC*_e_ (kg m^−3^) was computed by dividing *SSY* by *h*. The maximum instantaneous sediment concentration (*SC*_max_, kg m^-3^) was also used to represent the level of the sediment concentration of a single event.

The data we used are on a single storm basis. The runoff factors we examined include 4 factors: *h*, *q*_max_ (peak flow discharge normalised for drainage areas, m^3^ s^-1^ km^-2^), *hq*_max_ (the product of *h* and *q*_max_) and h+*q*_max_ (the sum of *h* and *q*_max_)_._ The use of h+*q*_max_ follows [23, 30]. The rainfall factors we examined include 12 factors: *P* (mm), *T* (rainfall duration, min), *I* (mean rainfall intensity, mm min^-1^), *I*_10_ (mm min^-1^)_,_ *I*_20_ (mm min^-1^), *I*_30_ (mm min^-1^), *EI*_10,_ *EI*_20_, *EI*_30_, *PI*_10,_ *PI*_20_ and *PI*_30_. The storm kinetic energy, *E* (J m^-2^), was calculated as follows:

$$E\;=\;\sum_{r=1}^{m}e_{r}p_{r}\;,$$ (1)

where *e*_r_ is the rainfall kinetic energy per unit depth of rainfall per unit area (J m^-2^ mm^-1^), and *p*_r_ is the depth of rainfall (mm) for the *r*th interval among *m* intervals of the storm hyetograph. *e*_r_ is calculated by an empirical equation building on measurements of the drop size distribution of 195 storms in the Loess Plateau [45]:

$$e_{r}=\;28.95\;+12.3\;log_{10}i_{r,}$$ (2)

where *i*_r_ (mm min^-1^) represents the mean rainfall intensity for the *r*th interval. After unit conversion, Equation (3) is almost identical to the rainfall intensity-energy equation of the USLE [4]. The discrepancy between the two equations is less than 10% for rainfall intensities from 1 to 40 cm h^-1^.

## Methodology

Assumed that *r*_1_ and *r*_2_ represent the correlation coefficients of any two rainfall factors with *SSY*. To compare *r*_1_ and *r*_2_, *Sinzot et al*. [12] and *Chen et al*. [19] used the following statistic:

$$Z=(z_{1}-z_{2})\sqrt{\frac{N-3}{2},}$$ (3)

where $z_{1}=\frac{1}{2}\ln\frac{1+r_{1}}{1-r_{1}}$ and $z_{2}=\frac{1}{2}\ln\frac{1+r_{2}}{1-r_{2}}$ are the Fisher z-transformed values for *r*_1_ and *r*_2_, *N is* the sample size. However, Equation (4) is applicable only to independent correlations [46] and cannot be used to compare *r*_1_ and *r*_2_ because they have a common dependent variable, *SSY*.

Meng’s tests [42] are widely applied for comparing correlations between a dependent variable and a set of independent variables. This study used these tests because they take a rather simple and thus easy-to-use form and perform as well as other statistical tests in terms of controlling the Type I error and power [41]. To compare *r*_1_ and *r*_2_, a *Z* (standard normal) test (termed “Meng’s Z_1_ test” in the following section for simplicity) is used [42]:

$$Z=(z_{1}-z_{2})\sqrt{\frac{N-3}{2(1-r_{x})H}},$$ (4)

where *r*_x_ is the correlation between the two rainfall factors under examination,

$$H=\frac{1-f\overset{-}{r^{2}}}{1-\overset{-}{r^{2}}},$$ (5)

$$f=\frac{1-r_{x}}{2(1-\bar{r^{2}})},$$ (6)

where $\bar{r^{2}}=(r_{1}^{2}+r_{2}^{2})/2$, and *f* should be set to 1 if the right term of Equation (7) is larger than 1.

If the comparison involves *k* rainfall factors (*k* > 2), the statistic (termed “Meng’s χ^2^ test” hereafter) used to test the heterogeneity of the correlations of the *k* factors with *SSY* is as follows [42]:

$$\;\chi^{2}(k-1)\;=\;\frac{(N-3)\;\sum_{i}{\left(z_{i}-\bar{z}\right)}^{2}}{(1-r_{x})H}\;,$$ (7)

where *z*_*i*_ is the Fisher z-transformed correlation coefficient for the *i*th rainfall factor (*i* ≤ *k*), and $\bar{z}$ is the mean of the *z*_*i*_ values. In the definition of *H* given by Equation (5), $\bar{r^{2}}$ becomes the mean of the $r_{i}^{2}$, and *r*_*x*_ becomes the median intercorrelation among the factors under testing. The resulting χ^2^ statistic is χ^2^ distributed on *k*-1 degrees of freedom.

By comparing a correlation with the average of the *k*-1 other correlations, *Meng et al*. [42] also designed a standard *Z* test (termed “Meng’s Z_2_ test” hereafter) to determine whether a contrast exists among the *k* factors under examination:

$$Z=r_{\lambda z}\sqrt{\chi^{2}(k-1)}$$ (8)

where r_λx_ represents the correlation coefficient between *z*_*i*_ and λ_i_. The values of λ_i_ are the contrast weights assigned to each *z*_*i*_. The sum of the λ_i_s must be zero. If we wish to determine whether the first factor differs among four factors in terms of their correlations with *SSY*, for instance, λ_i_s should be-3, 1, 1 and 1, respectively.

Based on Meng’s tests, we would use two methods to determine the rainfall-runoff erosivity indices. Method one repeatedly uses Meng’s Z_2_ test (Equation (8)) to remove factors that are poorly correlated with *SSY* in a stepwise way. Method two performs all paired comparisons using Meng’s Z_1_ test (Equation (5)). The Type I error, however, would increase when multiple comparisons are conducted simultaneously. We used Hochberg’s Sharpened Bonferroni correction to counteract the problem of multiple comparisons [47, 48]. Given a *p* value resulting from Meng’s Z_1_ test, the corrected value according to the Hochberg approach is *p*’ = *Rp*, where *R* is the rank value in descending order of the given *p* value among all obtained *p* values. The *p* values given below are one-tailed for the Meng’s χ^2^ test, and two-tailed for all other tests.

Using the two methods above, we would determine the rainfall erosivity index among the 12 rainfall factors and the runoff erosivity index among the 4 runoff factors. We limited our analyses of rainfall factors to the six experimental sites in the Tuanshangou subwatershed (#3 in Table 1 and the five plots in Table 2) due to the lack of reliable rainfall data at larger scale. A total of 222 events were used. Events without detailed hyetograph data were excluded. The analyses of runoff factors involve 379 events observed at all nine experimental sites listed in Tables 1 and 2.

## Results

### Method One—a stepwise procedure using Meng’s Z_2_ test

**Fig 1:**
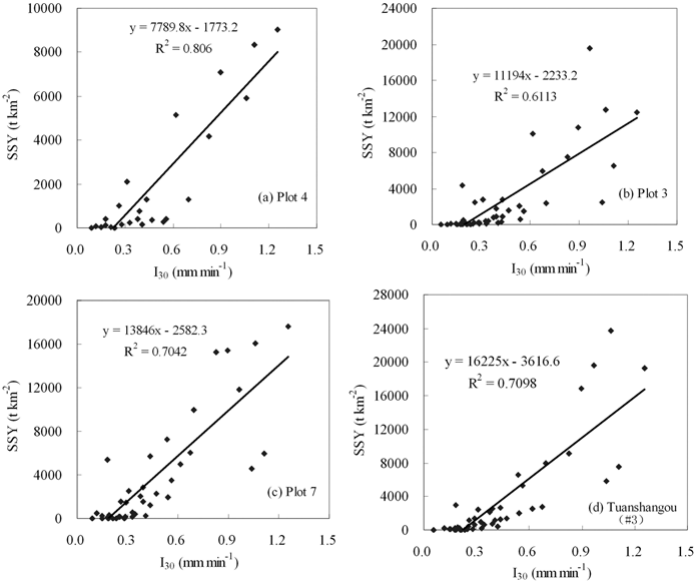
The relationship between *I*_30_ and *SSY* for the Tuanshangou station (#3) and three experimental runoff plots within it.

Table 3 presents the correlation coefficients between *SSY* and the 12 rainfall factors we examined. Factors other than *T* are generally well correlated with *SSY* (*p* < 0.01). The obtained correlation coefficients for *T*, *I* and *P* are much smaller than those for *I*_t_ (*I*_10,_ *I*_20_ and *I*_30_), *EI*_t_ (*EI*_10,_ *EI*_20_ and *EI*_30_) and *PI*_t_ (*PI*_10,_ *PI*_20_ and *PI*_30_). The relationship between *I*_30_ and *SSY* for four of the sites was plotted in Fig. 1.

**Table 3:** Correlation coefficients of the rainfall factors with *SSY* and *SC*_e_.^a^

	*SSY*						*SC*_e_ ^b^					
	Plot 4	Plot 2	Plot 3	Plot 7	Plot 9	#3	Plot 4	Plot 2	Plot 3	Plot 7	Plot9	#3
*I*_10_	**0.84**	**0.82**	**0.80**	**0.75**	**0.78**	**0.76**	0.44	0.16	0.23	0.35	0.39	**0.71**
*I*_20_	**0.89**	**0.88**	**0.80**	**0.81**	**0.82**	**0.82**	0.38	0.16	0.12	0.28	0.35	**0.62**
*I*_30_	**0.90**	**0.90**	**0.78**	**0.84**	**0.84**	**0.84**	0.32	0.09	0.02	0.16	0.25	0.44
*EI*_10_	**0.90**	**0.88**	**0.81**	**0.83**	**0.88**	**0.86**	0.19	0.04	0.12	0.18	0.20	0.37
*EI*_20_	**0.90**	**0.88**	**0.78**	**0.83**	**0.87**	**0.87**	0.17	0.02	0.08	0.13	0.17	0.30
*EI*_30_	**0.88**	**0.87**	**0.75**	**0.83**	**0.86**	**0.86**	0.12	-0.04	0.02	0.04	0.09	0.17
*PI*_10_	**0.81**	**0.82**	**0.75**	**0.81**	**0.84**	**0.82**	0.04	-0.04	0.10	0.16	0.14	0.24
*PI*_20_	**0.81**	**0.82**	**0.73**	**0.80**	**0.84**	**0.82**	0.03	-0.05	0.06	0.11	0.11	0.17
*PI*_30_	**0.79**	**0.81**	**0.69**	**0.80**	**0.82**	**0.81**	-0.01	-0.10	0.00	0.03	0.04	0.05
*T*	-0.13	-0.03	-0.01	0.02	0.00	-0.03	-0.48	-0.11	-0.02	-0.14	-0.15	-0.38
*I*	0.29	0.25	**0.55**	**0.40**	**0.55**	**0.50**	0.41	-0.03	0.10	0.30	0.27	0.50
*P*	**0.48**	**0.53**	**0.46**	**0.55**	**0.61**	**0.54**	-0.26	-0.18	-0.01	-0.01	-0.03	-0.15

^a^ Boldface denotes statistical significance at the 0.05 level. Meanings and units of the variables were specified in Section “Study area and data.” The same is for other tables.

^b^ Only major sediment-producing events were used to calculate the correlation coefficients.

To determine whether a rainfall factor represents a contrast, we performed 12 Meng’s Z_2_ tests at each of the six sites in the Tuanshangou subwatershed. The result (Test 1 in Table 4) shows that the correlation coefficients of *T*, *I* and *P* with *SSY* are significantly smaller (*p* < 0.005) than the average of factors other than itself at every site. Factors of *PI*_20_ and *PI*_30_ are not contrasts (*p* > 0.05) at any site. Hence, these five factors (*T*, *I*, *P*, *PI*_20_ and *PI*_30_) were excluded as candidates for the erosivity index. Meng’s Z_2_ tests of the seven remaining factors shows that *PI*_10_ at Plot 4 and *I*_10_ at Plot 7, Plot 9 and #3 are less correlated with *SSY* (Test 2 in Table 4; *p* < 0.02). When factors of *I*_10_ and *PI*_10_ were removed (Test 3 in Table 4), no contrast was detected among the five remaining factors of *I*_20_, *I*_30_, *EI*_10,_ *EI*_20_ and *EI*_30_ at all sites (*p* > 0.08). The same result holds when using Meng’s χ² test, which returns a quite high *p* value at the six sites (0.96, 0.92, 0.43, 0.90, 0.31 and 0.39, respectively), to examine the heterogeneity of the correlations of the five factors with *SSY*.

**Table 4:** *p* values resulting from Method One for the rainfall factors.^a^

Test^b^	Site	*I*_10_	*I*_20_	*I*_30_	*EI*_10_	*EI*_20_	*EI*_30_	*PI*_10_	*PI*_20_	*PI*_30_	*T*	*I*	*P*
1	Plot 4	0.12	**	**	**	**	**	0.46	0.44	0.76	**	**	**
	Plot 2	0.22	**	**	**	**	**	0.33	0.27	0.45	**	**	**
	Plot 3	**	**	**0.02**	**	**0.02**	0.15	0.14	0.44	0.94	**	**	**
	Plot 7	0.71	0.05	**	**	**	**	0.07	0.07	0.12	**	**	**
	Plot 9	0.94	0.12	**0.02**	**	**	**	**0.03**	0.05	0.13	**	**	**
	#3	0.87	0.07	**0.01**	**	**	**	0.09	0.07	0.12	**	**	**
2	Plot 4	0.16	0.75	0.38	0.25	0.24	0.81	**0.02**					
	Plot 2	0.12	0.67	0.12	0.58	0.48	0.86	0.06					
	Plot 3	0.46	0.40	0.90	0.24	1.00	0.18	0.20					
	Plot 7	**0.01**	0.76	0.30	0.51	0.41	0.46	0.62					
	Plot 9	**	0.33	0.92	0.10	0.15	0.33	0.87					
	#3	**	0.41	0.80	0.15	0.08	0.17	0.34					
3	Plot 4		0.67	0.90	0.69	0.69	0.62						
	Plot 2		0.80	0.38	0.89	0.98	0.61						
	Plot 3		0.41	0.79	0.23	0.90	0.11						
	Plot 7		0.33	0.62	0.94	0.80	0.87						
	Plot 9		0.08	0.44	0.25	0.36	0.68						
	#3		0.08	0.56	0.49	0.31	0.54						

^a^ Each *p* value corresponds to a Meng’s Z_2_ test and indicates whether the factor under examination can be considered as a contrast.

** denotes statistical significance at the 0.01 level. Boldface denotes statistical significance at the 0.05 level.

^b^ Test 1 involves all 12 factors we examined, Test 2 involves seven and Test 3 involves five of the factors. See Section “Method One” for details.

The four runoff factors of *h*, *q*_max_, *h+q*_max_ and *hq*_max_ are all highly correlated with *SSY* (*p* < 0.01) at all nine sites, from plots to watersheds (Table 5). The derived correlation coefficients between *h* and *SSY* are either at the maximum or simply slightly smaller than the maximum (generally < 0.01) at each site. Meng’s Z_2_ tests (Table 6) show that the correlation between *h* and *SSY* is significantly higher than the average of the three remaining factors at seven of nine examined sites (*p* < 0.0006). In contrast, the correlation between *q*_max_ and *SSY* is significantly lower than the average of the three remaining factors at eight sites (*p* < 0.031). Hence, *h* should be preferred to *q*_max_ as the predictive factor of *SSY*. The factor of *h*+*q*_max_ is better correlated with *SSY* than the average of the three remaining factors at four sites (*p* < 0.04), whereas the factor of *hq*_max_ is less correlated with *SSY* than the average of the three remaining factors at six sites (*p* < 0.02). This demonstrates that the combination of *q*_max_ with *h* would impair rather than improve the ability of *h* to predict *SSY*.

**Table 5:** Correlation coefficients of the runoff factors with *SSY* and *SC*_e_.^a^

Site	*SSY*				*SC*_e_ ^b^			
	*h*	*q*_max_	*h*+*q*_max_	*hq*_max_	*h*	*q*_max_	*h*+*q*_max_	*hq*_max_
Plot 4	**0.94**	**0.96**	**0.96**	**0.92**	0.33	0.41	0.39	0.26
Plot 2	**0.96**	**0.86**	**0.92**	**0.91**	-0.002	0.35	0.26	-0.06
Plot 3	**0.94**	**0.91**	**0.95**	**0.95**	0.04	0.21	0.17	0.21
Plot 7	**0.99**	**0.89**	**0.95**	**0.89**	-0.19	0.02	-0.06	-0.24
Plot 9	**0.99**	**0.9**	**0.96**	**0.91**	0.04	0.23	0.18	0.18
#3	**0.99**	**0.92**	**0.97**	**0.93**	0.22	**0.63**	**0.52**	0.44
#4	**0.99**	**0.92**	**0.99**	**0.95**	0.35	**0.55**	**0.45**	**0.41**
#9	**0.99**	**0.91**	**0.99**	**0.91**	0.20	0.30	0.22	0.20
#12	**0.97**	**0.84**	**0.98**	**0.94**	-0.07	0.24	-0.07	0.08

^a^ Boldface denotes statistical significance at the 0.05 level.

^b^ Only major sediment-producing events were used to calculate the correlation coefficients.

**Table 6:** *p* values resulting from Method One for the runoff factors.^a^

Site	*h*	*q*_max_	*h+q*_max_	*hq*_max_
Plot 4	0.32	0.19	**0.04**	***0*.*02***
Plot 2	**0.0005**	***0*.*007***	0.89	0.51
Plot 3	0.72	***0*.*031***	0.57	0.22
Plot 7	**0**	***4*.*9E-06***	0.84	***1*.*3E-06***
Plot 9	**0**	***1*.*5E-06***	0.96	***2*.*8E-05***
#3	**5.9E-09**	***1*.*7E-05***	0.10	***1*.*7E-03***
#4	**6.3E-07**	***1*.*3E-12***	**7E-10**	***5*.*0E-05***
#9	**4E-14**	***6*.*4E-15***	**3.8E-15**	***2*.*4E-14***
#12	**0.0006**	***2*.*9E-10***	**7.2E-05**	0.27

^a^Each *p* value corresponds to a Meng’s Z_2_ test and indicates whether the factor under examination is a contrast among the four runoff factors. Boldface denotes statistical significance at the 0.05 level. Non-italic boldface indicates a significantly higher correlation with *SSY* than the average of the three remaining factors. Italic boldface indicates a significantly lower correlation.

### Method Two—multiple Meng’s Z_1_ tests adjusted using the Hochberg approach

Meng’s Z_2_ tests used above have clearly demonstrated that *T*, *I* and *P* are inferior to other factors for application as the erosivity index. To reduce complexity, these factors are not considered in this section.

To test the significance of the difference between correlations of the nine rainfall factors of *I*_t_, *EI*_t_ and *PI*_t_ with *SSY*, we performed 36 pairwise comparisons using Meng’s Z_1_ test at each of six sites within the Tuanshangou subwatershed. The resultant *p* values, together with the *p*’ values after the Bonferroni correction, are presented in Table 7. Fifty-one among the 216 comparisons produced significant differences (*p* < 0.05) in the absence of the Bonferroni correction, with most (33) involving comparisons between *PI*_t_ and *EI*_t_. When the Bonferroni correction was performed, significant differences remained for only 9 comparisons (*p*’ < 0.04), all of which involved the comparison between *PI*_t_ and *EI*_t_. The results adjusted using the Bonferroni correction demonstrate that *EI*_t_ is better correlated with *SSY* than *PI*_t_ in some cases, and the correlations with *SSY* was not significantly different among six factors of *EI*_t_ and *I*_t_ (*p*’ > 0.11).

**Table 7:** *p* values resulting from Method Two for the rainfall factors.^a^

	Plot 4		Plot 2		Plot 3		Plot 7		Plot 9		#3	
	*p*	*p*’	*p*	*p*’	*p*	*p*’	*p*	*p*’	*p*	*p*’	*p*	*p*’
*I*_*10*_ *vs*. *I*_*20*_	0.08	1.91	**0.04**	1.2	0.92	2.75	**0.02**	0.6	**0.04**	1.4	**0.02**	0.5
*I*_*30*_	0.12	2.54	**0.04**	1.2	0.58	4.63	**0.02**	0.7	0.06	1.8	**0.03**	0.7
*EI*_*10*_	0.20	3.48	0.29	4.9	0.82	4.10	0.09	2.8	**0.01**	0.5	**0.01**	0.4
*EI*_*20*_	0.23	3.73	0.29	4.6	0.73	4.39	0.11	3.4	**0.04**	1.2	**0.02**	0.6
*EI*_*30*_	0.47	5.64	0.45	5.8	0.38	4.53	0.17	4.8	0.09	2.3	0.05	1.3
*PI*_*10*_	0.65	5.83	0.89	3.6	0.38	4.18	0.37	7.4	0.22	4.4	0.33	5.3
*PI*_*20*_	0.67	4.70	0.95	0.9	0.22	3.93	0.41	7.8	0.31	4.1	0.33	5.6
*PI*_*30*_	0.52	5.72	0.81	4.9	0.11	2.38	0.52	7.3	0.47	4.7	0.45	5.9
*I*_*20*_ *vs*. *I*_*30*_	0.44	5.76	0.14	3.0	0.17	3.48	0.08	2.6	0.23	4.3	0.13	2.8
*EI*_*10*_	0.65	6.46	0.95	1.9	0.83	3.33	0.53	6.9	0.09	2.3	0.15	2.9
*EI*_*20*_	0.65	5.24	0.88	4.4	0.62	4.31	0.50	7.5	0.15	3.3	0.13	2.8
*EI*_*30*_	0.97	1.94	0.90	2.7	0.25	4.05	0.60	7.2	0.31	4.4	0.26	5.0
*PI*_*10*_	0.18	3.40	0.25	5.0	0.28	4.18	0.92	2.8	0.67	4.0	0.94	1.9
*PI*_*20*_	0.19	3.46	0.29	4.4	0.12	2.62	0.92	3.7	0.81	3.2	0.99	1.0
*PI*_*30*_	0.14	2.71	0.24	5.0	0.05	1.23	0.81	6.5	0.97	1.9	0.87	3.5
*I*_*30*_ *vs*. *EI*_*10*_	0.87	3.47	0.53	6.4	0.37	4.79	0.80	7.2	0.23	4.2	0.43	6.0
*EI*_*20*_	0.86	4.32	0.60	6.6	0.93	1.85	0.88	4.4	0.28	4.3	0.32	5.7
*EI*_*30*_	0.72	4.31	0.44	6.1	0.43	4.30	0.86	6.0	0.50	4.0	0.50	4.5
*PI*_*10*_	0.08	1.85	0.07	1.7	0.52	4.66	0.41	7.1	0.97	1.0	0.50	5.0
*PI*_*20*_	0.08	1.78	0.08	1.8	0.22	3.79	0.41	7.4	0.82	2.5	0.56	3.9
*PI*_*30*_	**0.05**	1.33	0.06	1.6	0.08	1.73	0.35	7.3	0.62	4.3	0.46	5.5
*EI*_*10*_ *vs*. *EI*_*20*_	0.99	0.99	0.74	5.2	**0.01**	0.37	0.75	7.5	0.68	3.4	0.56	4.5
*EI*_*30*_	0.28	4.16	0.65	6.5	**	0.11	0.94	1.9	0.48	4.4	0.94	2.8
*PI*_*10*_	**	**	**0.01**	0.4	**0.01**	0.38	0.27	6.6	0.06	1.8	**0.02**	0.6
*PI*_*20*_	**	**0.03**	**0.03**	0.9	**	0.07	0.32	7.1	0.07	1.8	0.06	1.5
*PI*_*30*_	**	**0.03**	**0.03**	0.8	**	**0.02**	0.28	6.8	0.06	1.7	0.06	1.4
*EI*_*20*_ *vs*. *EI*_*30*_	0.07	1.73	0.27	4.9	**	0.13	0.87	5.2	0.40	4.8	0.48	5.3
*PI*_*10*_	**	**0.01**	**0.02**	0.5	0.19	3.62	0.21	5.6	0.09	2.3	**0.01**	0.3
*PI*_*20*_	**	**	**0.01**	0.4	**0.01**	0.38	0.18	5.1	**0.04**	1.3	**0.01**	0.4
*PI*_*30*_	**	**	**	0.2	**	**0.04**	0.15	4.4	**0.03**	1.0	**0.01**	0.4
*EI*_*30*_ *vs*. *PI*_*10*_	**0.01**	0.31	0.07	1.7	0.95	0.95	0.29	6.8	0.28	4.5	**0.04**	1.2
*PI*_*20*_	**	0.11	**0.05**	1.3	0.31	4.32	0.21	5.4	0.10	2.3	**0.03**	0.8
*PI*_*30*_	**	**	**	0.3	**0.01**	0.36	0.09	2.9	**0.02**	0.8	**0.01**	0.2
*PI*_*10*_ *vs*. *PI*_*20*_	0.93	2.78	0.71	5.7	**0.02**	0.56	0.97	1.0	0.47	5.1	0.71	4.3
*PI*_*30*_	0.38	5.39	0.68	6.2	**	0.13	0.67	7.3	0.26	4.5	0.78	3.9
*PI*_*20*_ *vs*. *PI*_*30*_	0.10	2.30	0.26	5.0	**	0.13	0.46	7.4	0.20	4.3	0.38	5.7

^a^ Each *p* value represents a paired comparison using Meng’s Z_1_ test. The *p*’ values represent those adapted using the Hochberg approach [47].

** indicates statistically significant differences at the 0.01 level. Boldface indicates statistically significant differences at the 0.05 level.

To compare the strength of correlations between *SSY* and the four runoff factors, we performed six Meng’s Z_1_ tests at each of nine sites within the Dalihe watershed. At seven sites, *h* is more correlated with *SSY* than *q*_max_ (*p* < 0.001, *p*’ < 0.01; Table 8), regardless of whether the Bonferroni correction was applied. Only at Plot 4 was the obtained correlation coefficient between *h* and *SSY* smaller than that between *q*_max_ and *SSY* (Table 5). This difference, however, was not statistically significant (*p* = 0.26, *p*’ = 0.52). A total of 18 comparisons at the nine sites were made between the correlations of *h* and the combined factors of *h* and *q*_max_ (i.e. *h+q*_max_ and *hq*_max_) with *SSY*. The Meng’s Z_1_ test suggested a significant difference for 11 among the 18 comparisons, and almost all (10) remain significant after the Bonferroni correction (Table 8). Among the ten comparisons, *h* is more correlated with *SSY* for nine (*p* < 0.007, *p*’ < 0.007) and less correlated for only one (*p* < 0.01, *p*’ < 0.01; #12). This again indicates that both *q*_max_ and its combination with *h* are inferior to the single factor of *h* for the *SSY* predictions.

**Table 8:** *p* values resulting from Method Two for the runoff factors.^a^

	Plot 4		Plot 2		Plot 3		Plot 7		Plot 9	
	*p*	*p*’	*p*	*p*’	*p*	*p*’	*p*	*p*’	*p*	*p*’
*h vs*. *q*_max_	0.26	0.52	**	**	0.15	0.61	**	**	**	**
* h+q*_max_	0.09	0.34	0.05	0.15	0.90	0.90	**	**	**	**
* hq*_max_	0.49	0.49	**0.01**	0.05	0.62	1.87	**	**	**	**

	**#3**		**#4**		**#9**		**#12**			
	*P*	*p*’	*p*	*p*’	*p*	*p*’	*p*	*p*’		
*h vs*. *q*_max_	**	**	**	**	**	**	**	**		
* h+q*_max_	**	**	0.47	0.47	0.77	1.54	** ^b^	** ^b^		
* hq*_max_	**	**	**	**	**	**	**	**		

^a^ *p* values directly result from Meng’s Z_1_ test and *p*’ values are corrected values using the Hochberg approach [47].

** indicates statistically significant differences at the 0.01 level. Boldface indicates statistically significant differences at the 0.05 level.

^b^ This test suggests that *h* is less correlated with *SSY* than *h+q*_max_ although the obtained correlated coefficients are almost the same (0.973 vs 0.975). Other significant results all suggest a stronger correlation for *h*.

## Discussion

### Rainfall factors

For rainfall factors, the results of two methods slightly differ: Method one suggested that *I*_20_, *I*_30_, *EI*_10,_ *EI*_20_ and *EI*_30_ are superior to other factors as a predictor of *SSY*; Method two excessively accepted *I*_10_ as an optimal predictor. This result may be related to the Bonferroni correction, which increases the likelihood of accepting the null hypothesis of identical correlations thereby increasing the risk of committing the type II errors [49].

The Loess Plateau is typically dominated by infiltration excess overland flows, and the runoff yield is determined by rainfall intensity rather than rainfall amount. In the Tuanshangou subwatershed, the median *T* is about 170 min. In contrast, the runoff duration at Plots 4, 2 and 3, with a median value of approximately 16 min, hardly exceeded 40 min. Rainfall during the low-intensity period is thus of little consequence to runoff yield and thus, to soil erosion. As a result, *P* is a poor indicator of *SSY*, as was also reported in [25, 40].

The single factor of *I*_30_, although equally as effective in predicting *SSY* as factors of *I*_20_, *EI*_10,_ *EI*_20_ and *EI*_30_, can be preferentially used as the rainfall erosivity index in practices because *I*_30_ is in form simpler than *EI*_t_ and can be measured somewhat more accurately than *I*_20_. *Wang* [16] also noted that the predictive ability of *EI*_t_ is only marginally higher than that of *I*_t_ in the Loess Plateau. The calculation of *E* involves data which are rarely available. Our finding shows that *E* is not necessarily included into the rainfall erosivity index thereby facilitating the obtainment of rainfall erosivity in the Loess Plateau.

Our calculations at six sites within the Tuanshangou subwatershed indicate that *I*_30_ summed over a year can explain 71 to 89% of the variation in yearly soil loss, an accuracy that is comparable to that of the use of the *EI*_*30*_ index in the USA [40]. Considering the fact that our data are from cropped plots, *I*_30_ can be directly applied to predicting soil loss in cropped areas, as opposed to the USLE, which first predicts erosion for the unit plot (bare fallow areas 22.1 m long on a 9% slope) and then predicts for the area of interest by introducing the topographic factors and the cover and management factors. Nevertheless, the mean of the annual cumulative *I*_30_ promises to act as the *R* factor of the USLE due to the sparse vegetation cover in the plots.

### Runoff factors

For runoff factors, the two methods present the same result: *h* is not only superior to *q*_max_ but is also superior to the combined factors of *h* and *q*_max_ as a predictor of *SSY*.

Similar to the rainfall case, flow discharge during flood events is primarily concentrated during the high-flow period, especially during the peak-flow period. Consequently, *q*_max_ correlates well with *h* (*r* > 0.77) and in turn, with *SSY* at every site (*r* > 0.83, Table 5). However, sediment concentrations at moderate discharges are more or less the same as those at high discharges in the Loess Plateau. Extremely high concentrations even primarily occur at low discharges from plots to watersheds (see Fig. 6 in [44] and Fig. 2 in [34]). This mismatch between sediment concentration and water discharge can be related to hyperconcentrated flows, which are well developed from upland slopes to river channels in the Loess Plateau [50, 51]. It is known that no direct relationship exists between sediment concentration and water discharge for hyperconcentrated flows [52]. In stream channels of the Chabagou watershed (#9), *SC*_max_ generally occur at flow discharges that are approximately 30–50% lower than *q*_max_ [53]. Hence, contrary to the rainfall case, *h* is more correlated with *SSY* than *q*_max_.

Runoff factors can provide better *SSY* predictions than rainfall factors in the Loess Plateau in terms of correlations (See Tables 3 and 5). The obtained correlation coefficients between *h* and *SSY* is larger than those between *I*_30_ and *SSY* at all six sites within the Tuanshangou subwatershed, and five of them being statistically valid (*p* < 0.02). Interrill erosion is closely related to rainfall factors, whereas rill erosion is primarily dependent on runoff factors [25, 54]. The higher correlation of runoff factors with *SSY* relative to rainfall factors can thus be linked to the dominance of rill erosion and the mass wasting over the interrill erosion in our study area.

### The best erosivity index

The rainfall-runoff factors we examined are generally inter-correlated. As a result, it can hardly be expected to improve the prediction accuracy by introducing more factors. *SSY* equals the product of *h* and *SC*_e_. There is no need to include factors that do not affect *SC*_e_ into the erosivity index if *h* has been included. We hereafter examine the correlations between the rainfall-runoff factors and *SC*_e_.

**Fig 2:**
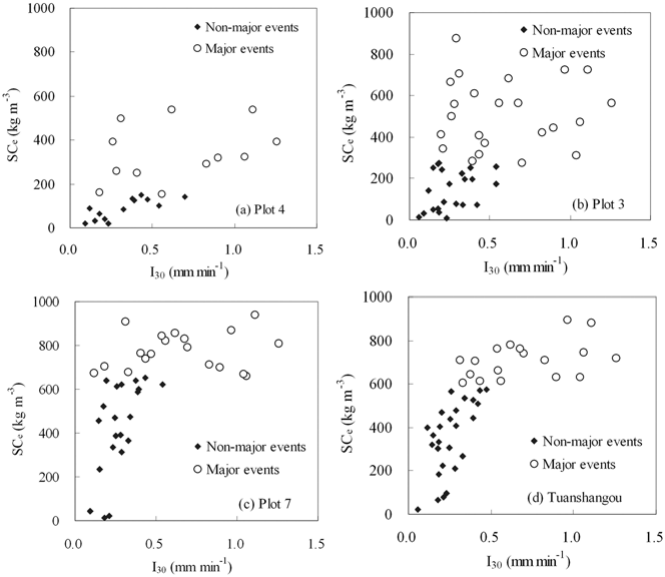
The relationship between *I*_30_ and *SC*_e_ for the Tuanshangou station (#3) and three experimental runoff plots within it.

**Fig 3:**
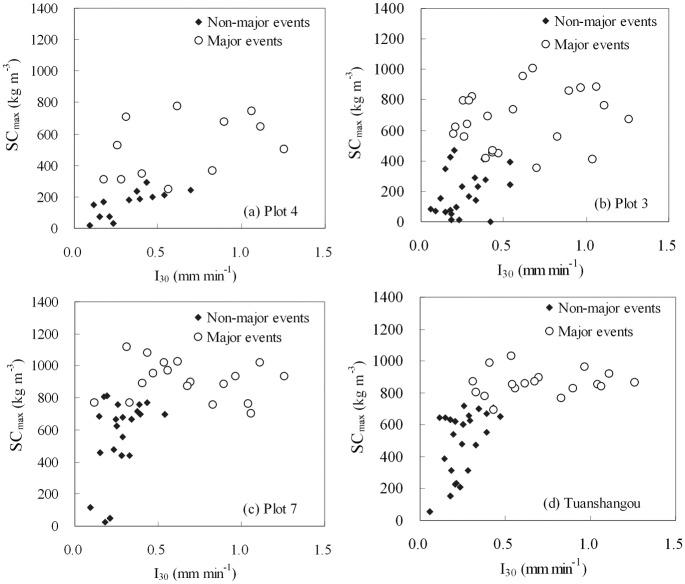
The relationship between *I*_30_ and *SC*_max_ for the Tuanshangou station (#3) and three experimental runoff plots within it.

Except for *T*, *I* and *P*, nine other rainfall factors correlate well with *SC*_e_ at all sites (*p* < 0.01). However, almost all of these correlations become insignificant with only two exceptions when only major sediment-producing events are considered (see Table 3). As in [35], we defined major sediment-producing events as high-concentrated events that accumulatively contribute 90% to the total sediment yield of all examined events. Scatter plots of *SC*_e_ and *I*_30_ at all sites are generally parallel to the x-axis for major sediment-producing events (Fig. 2), a result contrary to that observed by *Kinnel* [29] and *Chaplot et al*. [55]. The same observation holds for scatter plots of *SC*_max_ and *I*_30_ (Fig. 3).

**Fig 4:**
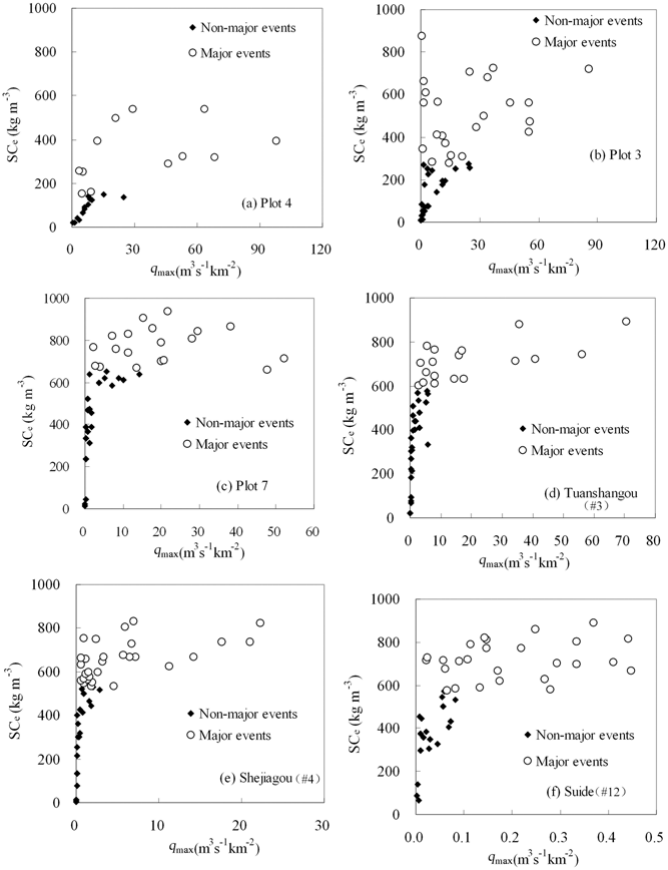
The relationship between *q*_max_ and *SC*_e_ for plots and watersheds within the Dalihe watershed

**Fig 5:**
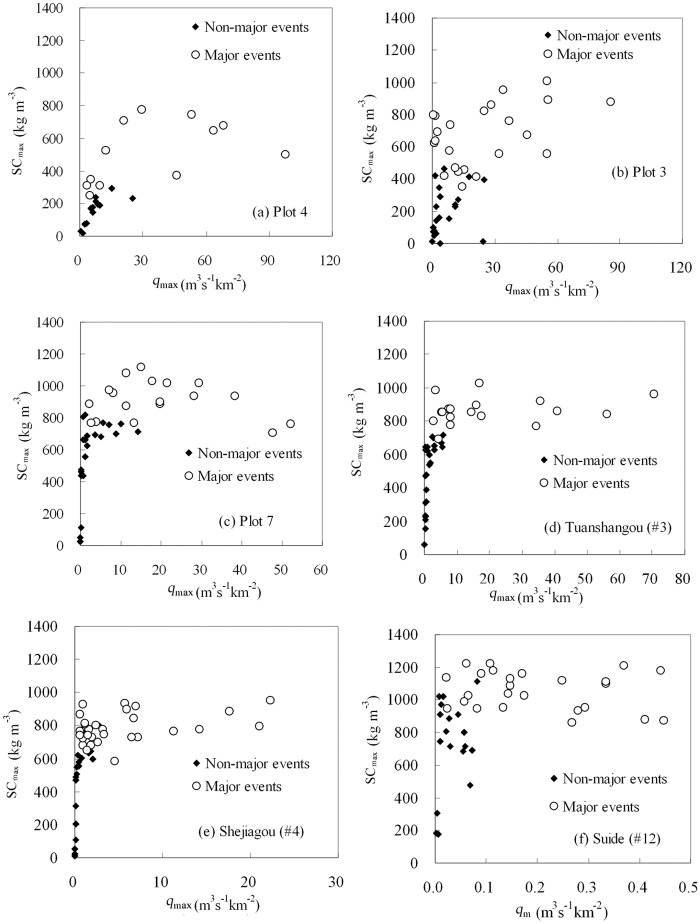
The relationship between *q*_max_ and *SC*_max_ for plots and watersheds within the Dalihe watershed.

For major sediment-producing events, *SC*_e_ and *SC*_max_ also remain independent of the four runoff factors although there are five exceptions among the 36 derived correlations (Table 5). Fig. 4 and Fig. 5 depict the relationships between *SC*_e_ and *q*_max_ and between *SC*_max_ and *q*_max_, respectively.

In terms of the correlation with *SSY*, the single factor of *h* is the best erosivity index among factors we examined at scales from plots to watersheds. It is not expected that the prediction accuracy would be further improved by introducing additional rainfall-runoff factors because these factors are ineffective in altering *SC*_e_ for major sediment-producing events. As was the case of *q*_max_, little was added to the prediction accuracy by combining *h* with *I*_30_. Among the six sites within the Tuanshangou subwatershed, *h*+*I*_30_ performs better than *h* only at one site (*p* = 0.03; Table 9), and *hI*_30_ is not as good as *h* at three sits (*p* < 0.046). When the runoff coefficient (*a*, given by *h* divided by *P*) was introduced, as did in the USLE-M [27], neither *aI*_30_ nor *aEI*_30_ provide better predictions of *SSY* than *h* at any site in terms of correlations. Moreover, *aI*_30_ and *aEI*_30_ are less correlated with *SSY* than *h* at three and two sites, respectively (*p* < 0.006; Table 9).

**Table 9:** Correlation coefficients between some combined factors and *SSY* and the comparisons with those between *h* and SSY.^a^

Site	correlation coefficients				*p* ^a^			
	*h*+*I*_30_	*hI*_30_	*aI*_30_ ^b^	*aEI*_30_ ^b^	*h*+*I*_30_	*hI*_30_	*aI*_30_ ^b^	*aEI*_30_ ^b^
Plot 4	0.94	0.95	0.93	0.95	0.26	0.58	0.67	0.42
Plot 2	0.96	0.94	0.93	0.94	0.22	0.22	0.11	0.16
Plot 3	0.95	0.94	0.95	0.94	0.41	0.76	0.80	0.95
Plot 7	0.99	0.96	0.94	0.95	**0.03**	***5E-07***	***3E-10***	***3E-08***
Plot 9	0.99	0.98	0.96	0.98	0.40	***0*.*006***	***4E-07***	***0*.*006***
#3	0.99	0.98	0.97	0.98	0.07	***0*.*046***	***0*.*002***	0.08

^a^ *p* values result from Meng’s Z_1_ test. Each *p* value represents a comparison between correlations of a combined factor and *h* with *SSY*. Boldface denotes statistical significance at the 0.05 level. Non-italic Boldface indicates that the combined factor is more correlated with *SSY* than *h*, whereas italic boldface indicates the combined factor is less correlated with *SSY* than *h*.

^b^ *a*, the runoff coefficient, is computed by dividing *h* by *P*.

## Conclusions

Based on Meng’s tests, this study presents two methods to determine the erosivity index among a number of rainfall-runoff factors by comparing their correlations with *SSY*. The first method involves a stepwise procedure to remove factors that are poorly correlated with *SSY*. The second method involves multiple comparisons that are adjusted using a Bonferroni correction. It appears that few studies have compared correlations on a statistical basis, not only within the soil erosion community but also within the entire geoscience community. Our methods therefore have wide significance, not only for determining the best predictor, but also in other respects, such as comparing model performance, which is often indexed by the correlation between observed and modeled values.

Using the methods described above, we determined the erosivity indices of rainfall and runoff in a typical Chinese Loess Plateau watershed. Among 12 rainfall factors under examination, *I*_20_, *I*_30_, *EI*_10,_ *EI*_20_ and *EI*_30_ were found to be the most correlated with *SSY* at scales from plots to subwatersheds (< 1 km^2^). We suggested the use of *I*_30_ as the rainfall erosivity index, although it is equally effective as the three remaining factors. The value of *I*_30_ summed over one year is also a good predictor of annual soil loss (*r* ^2^ > 0.7).

Runoff factors are more correlated with SSY than rainfall factors almost at all examined sites. Among the four studied runoff factors, h is correlated best with SSY at scales from plots to watersheds. Moreover, the combination of h with other rainfall-runoff factors, including rainfall intensity and peak discharge (as used in the MUSLE [24]), does not show enhanced ability to predict SSY compared with the single factor of h because these factors are of little importance in determining sediment concentration for major sediment-producing events. Introducing the runoff coefficient (as used in the USLE-M [27]) also added little to the prediction accuracy. Hence, we considered the single factor of h as the best erosivity index, although I30 would be useful in many cases considering the difficulty of measuring runoff.
